# Multiple Mechanisms Contribute to Lateral Transfer of an Organophosphate Degradation (*opd*) Island in *Sphingobium fuliginis* ATCC 27551

**DOI:** 10.1534/g3.112.004051

**Published:** 2012-12-01

**Authors:** Emmanuel Vijay Paul Pandeeti, Toshisangba Longkumer, Deviprasanna Chakka, Venkateswar Reddy Muthyala, Sunil Parthasarathy, Anil Kumar Madugundu, Sujana Ghanta, Srikanth Reddy Medipally, Surat Chameli Pantula, Harshita Yekkala, Dayananda Siddavattam

**Affiliations:** Department of Animal Science, School of Life Sciences, University of Hyderabad, Hyderabad, 500046, India

**Keywords:** integrative conjugative elements (ICE), genomic islands, catabolic transposons, phosphotriesterase (PTE), organophosphates

## Abstract

The complete sequence of pPDL2 (37,317 bp), an indigenous plasmid of *Sphingobium fuliginis* ATCC 27551 that encodes genes for organophosphate degradation (*opd*), revealed the existence of a site-specific integrase (*int*) gene with an attachment site *att*P, typically seen in integrative mobilizable elements (IME). In agreement with this sequence information, site-specific recombination was observed between pPDL2 and an artificial plasmid having a temperature-sensitive replicon and a cloned *attB* site at the 3′ end of the seryl tRNA gene of *Sphingobium japonicum*. The *opd* gene cluster on pPDL2 was found to be part of an active catabolic transposon with mobile elements *y4qE* and Tn*3* at its flanking ends. Besides the previously reported *opd* cluster, this transposon contains genes coding for protocatechuate dioxygenase and for two transport proteins from the major facilitator family that are predicted to be involved in transport and metabolism of aromatic compounds. A pPDL2 derivative, pPDL2-K, was horizontally transferred into *Escherichia coli* and *Acinetobacter* strains, suggesting that the *oriT* identified in pPDL2 is functional. A well-defined replicative origin (*oriV*), *repA* was identified along with a plasmid addiction module *rel*B/*rel*E that would support stable maintenance of pPDL2 in *Sphingobium fuliginis* ATCC 27551. However, if pPDL2 is laterally transferred into hosts that do not support its replication, the *opd* cluster appears to integrate into the host chromosome, either through transposition or through site-specific integration. The data presented in this study help to explain the existence of identical *opd* genes among soil bacteria.

Microbial metabolism of organophosphates (OP) attracted the attention of microbiologists as it contributes to the elimination of toxic OP insecticide residues from agricultural soils (Singh 2009). Microbial enzymes involved in degradation of OP compounds are divided into three major groups: organophosphate acid anhydrolases (OAA), phosphotriesteases, and methyl parathion hydrolases (Singh 2009). The OAAs were later shown to be prolidases involved in hydrolysis of the peptide bond of dipeptides with proline at their C terminus (Cheng *et al.* 1997; DeFrank and Cheng 1991; Vyas *et al.* 2010). Nerve agents like soman (GD; *O*-pinacolyl methylphosphonofluoridate), sarin (GB; *O*-isopropyl methylphosphonofluoridate), GF (*O*-cyclohexylmethylphosphonofluoridate), and tabun (GA; ethyl *N,N*-dimethylphosphoramidocyanidate) (Cheng *et al.* 1999) have been shown to be fortuitous substrates of prolidases (Cheng *et al.* 1999). Unlike the prolidases, no physiological substrate has yet been found for the other two OP hydrolyzing enzymes. Phosphotriesteases (PTE), encoded by the organophosphate degradation (*opd*) gene, are membrane-associated metalloenzymes and contain divalent Zn ions at their catalytic site (Benning *et al.* 2001; Mulbry and Karns 1989). The PTEs catalyze hydrolysis of the ester linkage found in structurally diverse groups of organophosphates, including nerve agents (Benning *et al.* 1994; Cheng and DeFrank 2000; Cho *et al.* 2004; Lai *et al.* 1995). The PTE purified from *Brevundimonas diminuta* hydrolyses parathion, the model OP compound, at a rate close to its diffusion limits and is considered to be an end point in enzyme evolution (Caldwell and Raushel 1991; Scanlan and Reid 1995). The OPs were recently (65 years ago) introduced into agriculture pest management, mainly as replacements for more persistent organochlorides (Davies *et al.* 1985). Therefore, evolution of PTEs in such a short period has become an interesting model in which to study molecular evolution (Elias and Tawfik 2012; Scanlan and Reid 1995). The studies conducted to date have shown quorum-quenching lactonases as progenitors of PTEs (Afriat *et al.* 2006; Elias and Tawfik 2012; Roodveldt and Tawfik 2005).

The third prominent organophosphate-degrading enzymes are methyl parathion hydrolases (MPH), encoded by the methyl parathion degradation (*mpd*) gene. The MPH enzymes known to date have been purified from *Pseudomonas* strains isolated from OP-polluted Chinese agricultural soils (Liu *et al.* 2005; Zhang *et al.* 2006; Zhongli *et al.* 2001). Despite having functional similarity, MPH enzymes share no homology with PTEs, indicating the existence of structurally independent organophosphate-degrading enzymes among soil bacteria (Dong *et al.* 2005). Unlike organophosphate hydrolase (OPH), the MPH has been shown to be a structural homolog of β-lactamases, the enzymes that confer resistance to β-lactam–derived antibiotics (Tian *et al.* 2008). Interestingly these two structurally different enzymes have an identical active site structure (Dong *et al.* 2005). This type of functional convergence pointed to the existence of independent paths in the evolution of organophosphate-hydrolyzing enzymes. Lateral transfer of *mpd* genes became evident with the discovery of identical *mpd* genes among bacterial strains isolated from OP-polluted Chinese soil samples (Zhang *et al.* 2006), and the *mpd* genes were shown to be part of an active transposon (Wei *et al.* 2009).

Identical *opd* genes have been found in soil bacteria isolated from diverse geographical regions. The dissimilar *opd* plasmids pCMS1 and pPDL2 were isolated from *B. diminuta* (pCMS1) and *Flavobacterium* sp. ATCC 27551 (pPDL2) that were enriched, respectively, from soil samples collected from agricultural fields of Texas, USA, and the International Rice Research Institute (IRRI), Philippines. They were shown to contain identical *opd* genes (Mulbry *et al.* 1986; Pandeeti *et al.* 2011), and a 7 kb region around the *opd* gene apparently constituted the only identity between these two dissimilar plasmids (Mulbry *et al.* 1986; Pandeeti *et al.* 2011; Siddavattam *et al.* 2003). However the sequence of the *opd* gene cluster in the self-transmissible pCMS1 showed no features of a transposable element (Pandeeti *et al.* 2011), and although a transposon-like organization was found in pPDL2, the *opd* cluster of pPDL2 has not been shown to be an active transposon (Siddavattam *et al.* 2003).

In the present study, we report the complete sequence of pPDL2, and we show experimentally that pPDL2 is a mobilizable plasmid having the capability to spread the *opd* cluster laterally by functioning both as an integrative mobilizable element and as an active transposon.

## Materials and Methods

### Strains and plasmids

Bacterial strains and plasmids used in the present study are shown in Table 1. Oligonucleotide primers used while performing PCR reactions are listed in supporting information, Table S1. *E. coli* and *Acinetobacter* sp. DS002 cells were grown in LB medium at 37° and 30°, respectively. The cultures of *Sphingobium fuliginis* ATCC 27551 were grown in modified Wakimoto medium at 30°. When necessary, the antibiotics ampicillin (100 μg/ml), tetracyclin (30 μg/ml), gentamycin (20 μg/ml), and kanamycin (30 μg/ml) supplemented the culture medium. The LB sucrose plates were prepared by adding 5% sucrose to LB agar plates. *Acinetobacter* sp. DS002 strains were grown in M9 medium containing filter-sterilized benzoate (5 mM) as a carbon source. Exconjugants of *Acinetobacter* sp. DS002 strains were selected on M9 agar plates supplemented with kanamycin.

**Table 1:** Bacterial strains and plasmids

Strain or Plasmid	Genotype or Phenotype	Reference or Source
*E. coli* DH5α	*supE*44 *ΔlacU*169 (Ø80 *lacZ ΔM*15) *hsdR*17 *recA*1 *endA*1 *gyrA*96 *thi*1 *relA*1	Hanahan (1983)
*E. coli* BL21 DE3	*hsdS gal(λcIts857 ind1 sam7 nin5lac UV5 T7 gene 1*	Studier and Moffatt (1986)
*E*. *coli* EC100D *pir*^+^116	*F*^-^ mcrA Δ*(mrr^-^hsdRMS^-^mcrBC) ϕ80dlacZΔM15 ΔlacX74 recA1 endA1 araD139 Δ(ara*, *leu)*7697 *galU galK λ^-^ rpsL (StrR) nupG pir-116(*DHFR*)*	Epicentre Biotechnologies, USA
*E. coli* HB101	*F^-^ mcrB mrr hsdS20(r_B_^-^ m_B_^-^) recA13 leuB6 ara-14 proA2 lacY1 galK2 xyl-5 mtl-1 rpsL20(Sm^R^) glnV44 λ^-^*	Boyer and Roulland-Dussoix (1969)
*E. coli* S17-1	*thi pro hsdR hsdM recA* RP4 2-Tc::Mu-Kn^r^::Tn7(Tp^r^ Sp^r^ Sm^r^)	Simon *et al.* (1983)
*Sphingobium fuliginis* ATCC 27551	Wild type strain, Sm^r^, Pm^r^, OPH^+^	Kawahara *et al.* (2010)
*Acinetobacter sp*.DS002	Cm^r^, Sm^r^, Ben^+^	Unpublished results from our lab
pRSET-A	Amp^r^, expression vector	Invitrogen, USA
pBluescript KS(II)	Amp^r^, *lacZ*^+^, cloning vector	Fermentas, USA
pET-23b(+)	Amp^r^, expression vector	Novagen, USA
pMMB206	Cm^r^, a low copy number broad host range expression vector	Morales *et al.* (1991)
pJQ210	Gm^r^, *sacB*^+^	Quandt and Hynes (1993)
pKD46	Amp^r^, Red recombinase expression plasmid	Datsenko and Wanner (2000)
pRK2013	Kmr, ColE1-based restricted-host-range helper plasmid	Ditta *et al.* (1980)
pSM3	Amp^r^, Tc^r^, *opd* gene replaced with *opd*::*tet in* plasmid pSM2	Siddavattam *et al.* (2003)
pPDL2	*opd*^+^, 37.3Kb indigenous plasmid	Mulbry *et al.* (1986)
pPDL2-K	*opd*^+,^ Km^r^, pPDL2 having single minitransposon EZ-Tn*5*<R6Kγ*ori*/KAN-2> insertion	This study
pPDL2-KT	*opd*^+,^ Km^r^, Tet^r^, pPDL2-K with replacement of *opd* with *opd*::*tet*	This study
pP4I	*oriT* sequence of pPDL2 cloned in pBluescript KS(II)	This study
pSFT1	*repB* gene cloned in pRSET-A as *Bam*HI and *XhoI* fragment	This study
pSFT2	Tc^r^, Mini replicon generated by ligating *oriV* and *repA* region of pPDL2 to *opd*::*tet* cassette	This study
pSDP7	Amp^r^, temperature sensitive cloning vector	This study
pSDP8	Amp^r^, *attB* sequence cloned in pSDP7 as *Hind*III fragment	This study
pSDP9	*ligA* and *ligB* genes cloned in pET-23b(+) as *NdeI* and *Hind*III fragment	This study

### Isolation and rescue cloning of pPDL2 from *Sphingobium fuliginis* ATCC 27551

Large indigenous plasmids of *Sphingobium fuliginis* ATCC 27551 were isolated by following the Currier Nester protocol with minor modifications (Currier and Nester 1976; Pandeeti *et al.* 2011). The plasmid preparation made from *Sphingobium fuliginis* ATCC 27551 was directly used for tagging with minitransposon EZ-Tn*5*<R6Kγori/KAN-2> using the EZTn5 <R6Kγ*ori*/KAN-2> insertion kit (Epicenter Biotechnologies, USA) following the manufacturer’s protocols. The isolated plasmid preparation and minitransposon was taken in equimolar concentrations and incubated with transposase for 2 hr at 37° to complete *in vitro* transposition. After 2 hr, transposase was inactivated by adding 1 μl of 1% SDS before incubating the reaction mixture for 10 min at 70°. The reaction mixture was then electroporated into *E. coli* EC100D *pir*-116 cells by setting the electroporator (Gene Pulser, Bio-Rad Laboratories, USA) at 2.5 kV, 200Ω for 4.5 msec. After electroporation, the cells were plated on LB plates supplemented with kanamycin to select transformants. Colonies having plasmid pPDL2 were identified by performing colony PCR using *opd* specific primers. The colonies that gave amplification of the *opd* gene were used to isolate plasmids having the minitransposon Tn*5*<R6Kγori/KAN-2>. The derivatives of plasmid pPDL2, named pPDL2-K, were then isolated from *E. coli pir*-116 cells using the BAC isolation protocol (Sambrook *et al.* 1989). The restriction profile of pPDL2-K was generated by digesting with *Sma*I, *Bam*HI, *Pst*I, *Sal*I, *Xho*I, *Hind*III, and *Eco*RI, and it was compared with the restriction profile of pPDL2 (Mulbry *et al.* 1987) to identify the exact point of minitransposon insertion. Further, all fragments of pPDL2-K obtained after digestion with *Pst*I and *Eco*RI were ligated independently into pBluescript (II) KS (+) digested with similar enzymes.

### Generation of the complete pPDL2 sequence

DNA sequencing was performed by using BigDye Terminator v1.1 Cycle Sequencing Kit (Applied Biosystems, USA), and an ABI PRISM 3100 Genetic Analyzer was used following manufacturer’s protocol. Either pPDL2 derivatives or subclones generated by cloning *Eco*RI and *Pst*I fragments were used as templates. Sequence reactions were generated using transposon-specific sequence primers when pPDL2 derivatives were used as templates, whereas universal forward and reverse primers were used to generate sequence from subclones. To fill gaps, a primer-walking strategy was employed. If the cloned fragment size was large, it was digested with different restriction enzymes and subcloned into pBluescript (II) KS(+) and were used for sequencing.

### Sequence assembly and annotation of pPDL2

All sequences were viewed and edited to remove vector sequences by using Chromas 2.13 software (www.technelysium.com.au/chromas). Sequences were assembled into contigs by using the program Contig Express of VectorNTI software (Invitrogen Technologies, USA). The assembled sequence of plasmid pPDL2 was annotated using Artemis sequence annotation tool (http://www.sanger.ac.uk/resources/software/artemis/) (Rutherford *et al.* 2000). The open reading frames (ORFs) were identified by using the built-in tool of the Artemis software, and the start codons in the predicted ORFs were fixed with the help of BLAST searches. BLAST searches were made against the nonredundant database of NCBI using BLAST program (www.ncbi.nlm.nih.gov/BLAST). IS elements, transposons, and their repeat elements were identified by doing a pairwise alignment using BlastN program against the ISfinder database (http://www-is.biotoul.fr/).

### Prediction of origin of replication (*oriV*)

The *oriV* was predicted based on sequence homology to other annotated or predicted *oriV*s and by performing GC skew analysis (Grigoriev 1998). While calculating the GC skew, the ratio of (G-C) to (G+C) was calculated per each window of equal length split over the total sequence of pPDL2. The GC skew was multiplied by w/c to find its dependence on subsequence length, where w and c are lengths of the subsequence and total sequence of pPDL2, respectively. As maximum and minimum value of GC skew is usually associated with termination and origin of replication (Frank and Lobry 1999), a similar approach was followed to predict *oriV* of plasmid pPDL2 origin.

### Prediction of *attP* sites

For predicting *attP* sites, two independent approaches were used. In the first approach, sequences of all available plasmids having identical integrases were collected from the NCBI database. From bacteria having these plasmids, a dataset of all tRNA sequences along with their upstream and downstream sequences was created. Pairwise alignments were made between the plasmids and the tRNA sequences to identify the *att* sites.

In a second approach, the genome sequences of bacteria having plasmids with integrase genes were collected from NCBI database. These genomic sequences were then used to predict genomic islands (GI) using Islandviewer software (http://www.pathogenomics.sfu.ca/islandviewer/query.php). The predicted GI sequences were then used to make pairwise alignments with sequences of tRNA genes of *Sphingomonas wittichii* and *Sphingobium japonicum*. Short sequence repeats that exactly matched the 3′ end of tRNA sequences were taken as putative *att* sites. The predicted *att* sites with a low E-value were considered as potential *att*P and *att*B sites. The *att* sites predicted in this manner were then used to align with plasmid pPDL2 sequence using BlastN to identify *attP* homologs. Alternatively, tRNA gene sequences taken from *S.wittichii* and *S. japonicum* were directly used to align with the pPDL2 sequence using BlastN to find short sequences that perfectly matched the 3′ end of tRNA genes.

### Bacterial conjugation experiments

Biparental mating experiments were done using *E. coli pir*-116 (pPDL2-K) as donor and *Acinetobacter* sp. DS002 as recipient. The triparental mating experiments were performed by including *E. coli* strain HB101 (pRK2013) as a helper. Biparental and triparental experiments were performed following standard protocols described elsewhere (Figurski and Helinski 1979). The exconjugants were selected by plating on selection plates having minimal media supplemented with 5 mM benzoate and kanamycin. Parent strains treated in a similar manner were plated on selection plates and served as negative controls.

### Cloning of synthetic *attB* site in a temperature-sensitive vector

A temperature-sensitive plasmid vector was constructed by using vectors pMMB206 (Morales *et al.* 1991) and pKD46 (Datsenko and Wanner 2000) as source plasmids. The temperature-sensitive replicative origin along with the *bla* gene were amplified as an *Eco*RV/*Sma*I fragment by using primers DSF005 and DSF006 as forward and reverse primers. The multiple cloning site and *lac* reporter system were taken from plasmid pMMB206 by digesting it with *Eco*RV/*Dra*I. These two fragments were then ligated to give plasmid pSDP7. The seryl tRNA with an *attB* sequence was synthesized before cloning it in pSDP7. Equal concentrations of the overlapping primers DSF001 and DSF002 were placed in a PCR tube in boiling water and left until the water reached room temperature. The contents were then brought down by briefly spinning, and then 2.5 μmol dNTP, a unit of *pfu*, and 10 μl 10× pfu buffer were added. The contents were made up to 100 μl with MilliQ water before incubating the tube at 72° for 10 min. An aliquot of 10 μl of the reaction mixture was then taken as a template to amplify *attB* by performing PCR using the primer set DSF003/DSF004 with *Hind*III recognition sites at the 5′ end. The amplicon was then digested with *Hind*III and cloned into pSDP7 to give pSDP8.

### *In vivo* integration assay

Plasmid pSDP8 was transformed into *E. coli pir*-116 (pPDL2-K), and the resultant cells were grown at 30° for 12 hr in LB medium with kanamycin and ampicillin. After incubation, cells were collected from 1 ml of culture and thoroughly washed before reinoculating them in fresh LB medium containing only kanamycin. The cultures were then grown for a further period of 12 hr at 37° to inhibit the replication of the temperature-sensitive plasmid pSDP8. After incubation, the culture was serially diluted, and appropriate dilutions were plated on LB agar with kanamycin and ampicillin. Plasmids were isolated from the resultant colonies, and the formation of a cointegrate due to site-specific recombination between *attP* and *attB* was determined by performing restriction analysis. The *att*B/P and *att*P/B sites were identified by DNA sequencing using universal forward and reverse primers.

### Transposition assay

For the transposition assay, we created a pPDL2-K derivative by replacing the *opd* gene with *opd*::*tet* through homologous recombination. *E. coli pir*-116 (pPDL2-K) was first transformed with the temperature-sensitive plasmid pKD46, which codes for red recombinase, in the presence of arabinose (Datsenko and Wanner 2000). The mid log-phase cultures obtained by growing in LB medium with 1 mM arabinose were made electrocompetent and immediately used to electroporate with insertionally inactivated *opd* (*opd*::*tet)* taken as a *Pst*I fragment from pMS3 (Siddavattam *et al.* 2003). The tetracycline-resistant colonies were then used to perform colony PCR using *opd*-specific primers. The colonies identified as carrying *opd*::*tet* were grown on them at 37° to eliminate temperature-sensitive pKD46. The ampicilin-sensitive, tetracycline-resistant colonies were then used to isolate the pPDL2-K derivative pPDL2-KT and to confirm the presence of *opd*::*tet* by restriction analysis.

The transposition assay used in the present study is described elsewhere (Siddavattam *et al.* 2003), except that the *sacB* containing pJQ210 (Quandt and Hynes 1993) was used as a reporter plasmid and plasmid pPDL2-KT served as donor of *opd* cluster. *E. coli* (pJQ210) cells were made electrocompetent and plasmid pPDL2-KT was electroporated following standard protocols (Dower *et al.* 1988). The electroporated cells were then grown in LB medium having no antibiotics for 2 hr. The cells were then plated on LB plates having gentamycin, sucrose, and tetracyclin. As plasmid pPDL2-KT requires a *pir* background, the tetracycline-resistant colonies found on sucrose plates were considered to have been generated due a transposition event disrupting the *sacB* gene. These colonies were then used to isolate plasmid and to analyze junction sites by performing restriction profile and PCR amplification. The PCR amplification was done by using *sacB*-specific primers (DSF0015/DSF0016) and primers (DSF0017/DSF0018) designed using pPDL2 sequence found upstream of the predicted Tn*3*-specific terminal repeats.

### Interactions between *oriT* and Mob protein

The predicted *rep*B gene downstream of *oriT* was amplified using a primer set DSF009/DSF0010 appended with *Bam*HI and *Xho*I sites. The amplicon was then cloned in expression vector pRSETA as a *Bam*HI*/Xho*I fragment to generate recombinant plasmid pSFT1. *E. coli* BL21 (pSFT1) cells were induced to express RepB_C-6His_ and affinity purified following procedures described elsewhere (Pandey *et al.* 2009). A 250 bp DNA fragment containing the *oriT* region was amplified using a primer pair (DSF0019 and DSF0020) designed by taking the sequence flanking *oriT* of pPDL2. The PCR amplicon containing *oriT* was end-labeled with [γ-^32^P] ATP using T4 polynucleotide kinase following established protocols (Sambrook *et al.* 1989). While performing mobility shift assay, the pure labeled *oriT* (2 pmol) was taken in 20 μl of binding buffer [20 mM Tris-HCl (pH 8.0), 1.0 mM EDTA, 6 mM MgCl_2_, 50 mM KCl, 50 µg/ml BSA, 5% (w/v) glycerol, 5 μg/ml of herring sperm DNA] and incubated for 20 min at 25° with various concentrations (0 ng, 100 ng, 250 ng, 500 ng and 1000 ng) of RepB_C-6His_. The reaction mixture was then resolved on a 5% (w/v) native polyacrylamide gel, and the mobility of labeled *oriT* was captured on X-ray film by performing autoradiography. Control reactions were performed either by omitting RepB_C-6His_ or by including increased concentrations (0.2 to 2 pmol) of cold *oriT*.

### Expression of the *lig* operon

The *lig* operon comprising the *ligB* and *ligA* genes was amplified from plasmid pPDL2 using primer set DSF0013/DSF0014 appended with *Nde*I-*Hind*III, respectively. The *Nde*I site in the forward primer corresponds to the 5′ region of *ligB* and overlaps with its start codon. Similarly, the reverse primer designed by taking the 3′ end of *ligA* contains a *Hind*III site created by changing the stop codon. The amplicon containing the *ligBA* genes was then cloned in pET23b as an *Nde*I/*Hind*III fragment to generate plasmid pSDP9. The cloning strategy followed to generate pSDP9 places both *ligB* and *ligA* under the transcriptional and translational signals of the expression vector. As the stop codon of *ligA* was eliminated to facilitate fusion of *ligA* with the vector-coded histidine tag, the LigA alone was expressed with C-terminal histidine tag. As active protocatechuate dioxygenease contained both LigB and LigA subunits, affinity purification using Ni-affinity column was expected to yield LigBA complex. Expression of LigBA was monitored both by measuring dioxygenease activity (Ono *et al.* 1970) and by detecting LigA_C-6His_ from Western blots using anti-his antibody (Pandey *et al.* 2009).

### Enzyme assays

Protocatechuate-4,5-dioxygenase assay was performed using purified LigBA complex. The reaction mixture contained protocatechate (20 µM) and 5 µg of purified LigBA complex in 100 mM Tris acetate buffer (pH 7.2). Formation of the ring clevage product, 2-hydroxy-4-carboxymuconic semialdehyde, was measured spectroscopically following established procedures (Ono *et al.* 1970). Similarly, formation of 2-hydroxymuconic semialdehyde was monitored at 375 nm when catechol was used as a substrate (Nozaki *et al.* 1970). The organophosphate hydrolase (OPH) assay was performed using methyl parathion as substrate. The OPH activity was measured by estimating the concentration of *p*-nitrophenol (Pakala *et al.* 2007). Specific activity of enzymes was expressed as micromoles of product formed per minute per milligram of protein.

## RESULTS

*Flavobacterium* sp. ATCC 27551 was one of the first organophosphate-degrading bacteria isolated from the agricultural soils of the International Rice Research Institute, Manila, Philippines (Sethunathan and Yoshida 1973). Recently it was reclassified as *Sphingobium fuliginis* ATCC 27551 (Kawahara *et al.* 2010). The OP degradation property has been shown to be encoded on one of its four indigenous plasmids, pPDL2 (Mulbry *et al.* 1986), the other three not being involved in this activity. Lateral gene transfer of *opd* is evident due to the existence of identical *opd* genes in bacterial strains belonging to different taxonomic groups. However, no systematic studies are available to explain the mechanism of lateral transfer of the *opd* gene cluster. Because pPDL2 contains a transposon-like *opd* cluster, we have taken it as a model system to study horizontal gene transfer (HGT) of the *opd* cluster. Initially pPDL2 was isolated from the other indigenous plasmids in *S. fuliginis* ATCC 27551 by performing rescue cloning. After *in vitro* transposition using the minitransposon Tn5 <R6Kγori-Kan2>, a pPDL2 derivative (pPDL2-K) having a single minitransposon insertion was identified by colony PCR using *opd* gene-specific primers.

**Figure 1:**
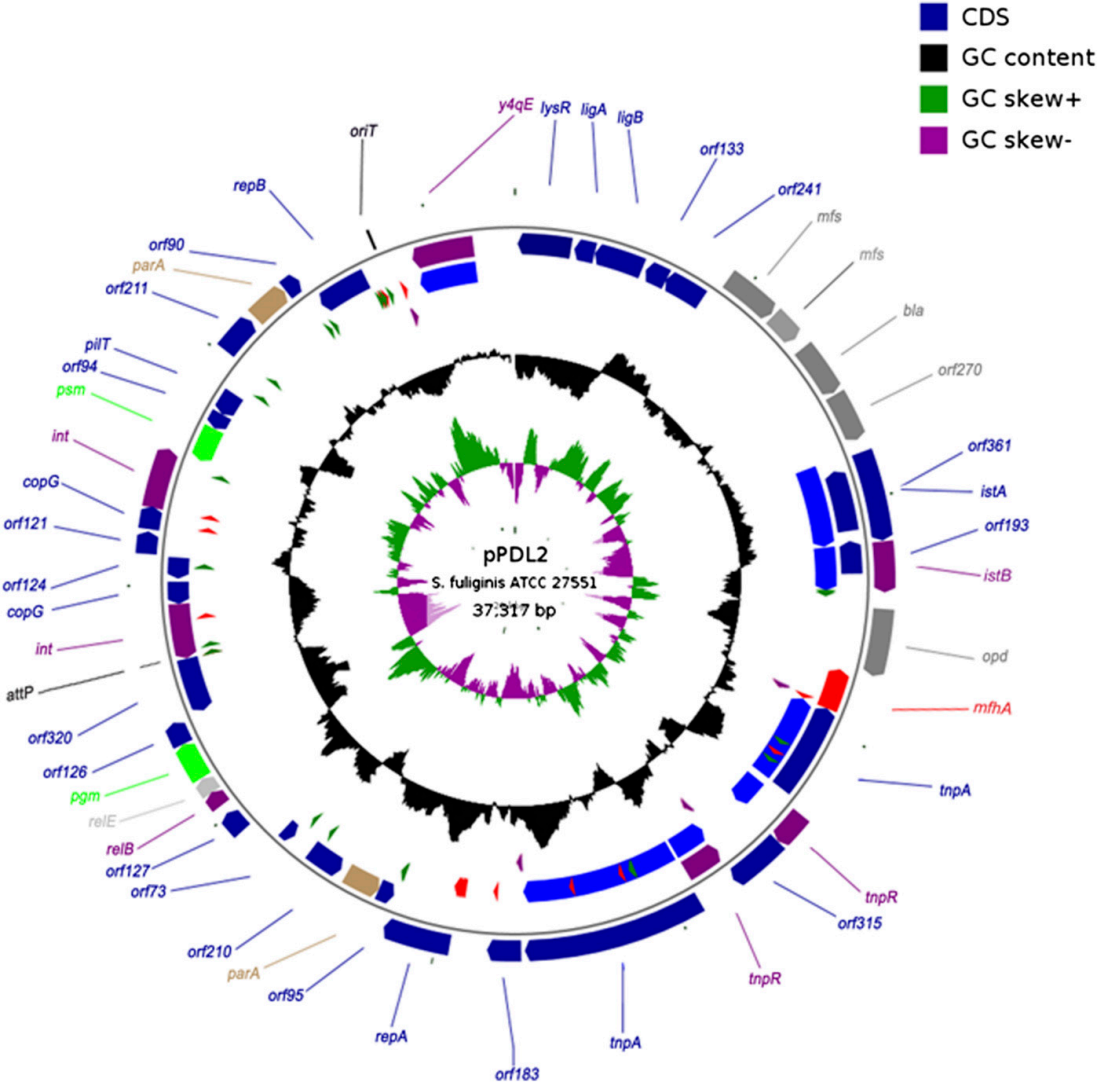
Physical map of plasmid pPDL2. Outer and inner circles indicate proteins encoded by sense and anti-sense strands, respectively. Third circle indicates mobile elements and repeat sequences. Direct (DR) and Inverted (IR) repeats are shown with filled red and green triangles, respectively. Tn*3*-specific repeats appear with filled purple triangles in the fourth circle. The fifth circle shows GC content across the plasmid sequence. The sixth and seventh circles represent GC-skew in sense and anti-sense strands.

The complete nucleotide sequence of 37,317 bp plasmid pPDL2 is deposited in GenBank (accession no. JX31–2671). Its GC content (62.37%) is very similar to the GC content of indigenous plasmids pCHQ1 (63%), pUT1 (63.7%), and pUT2 (61.0%) found in *S. japonicum* (Nagata *et al.* 2010, 2011). The GC content, GC skew, and the length of the plasmid indicates multiple changes in the sequence of pPDL2 either due to acquisition of foreign DNA or rearrangement of its own sequences (Figure 1). A total of 48 protein-coding regions were annotated, which represents a coding density of 91.3% (Table S2). Out of the 48 *orfs*, 18 of them were annotated as ORFs coding for hypothetical proteins, and 7 of them code for either transposases or associated resolvases (Table S2). Based on comparison with database entries, the ORFs coded by plasmid pPDL2 can be divided into a number of functional modules. Prominent among them are modules for replication and partition, mobilization, integration, and OP degradation, as well as mobile genetic elements.

### Replication initiation proteins

**Figure 2:**
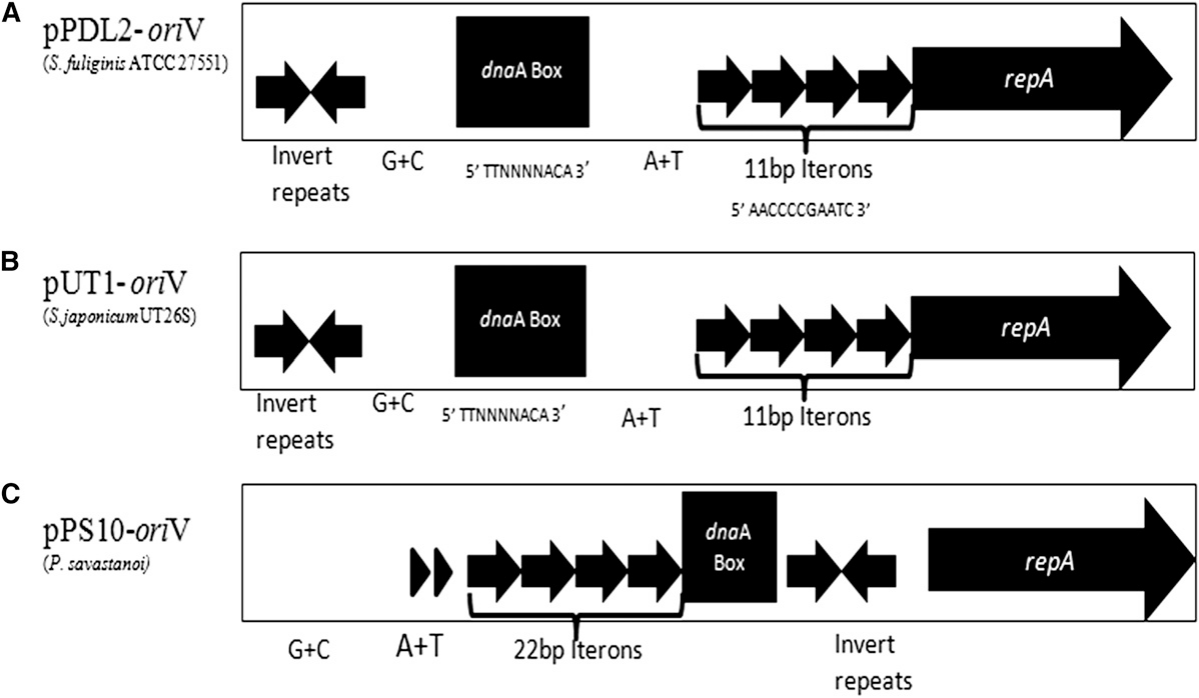
Replication module. Comparison of replicative origin (*ori*V) of plasmid pPDL2 (panel A) with *ori*V sequences of plasmids pUT1 (panel B) and pPS10 (panel C). Solid inverted arrows indicate inverted repeat sequences. The *dnaA* Box and iterons are shown with solid box and tandem arrows, respectively. The transcription orientation of *repA* is shown with a solid arrow. Filled triangles indicate repeats found in the A+T-rich region of pPS10.

In pPDL2, two ORFs, *orf22* and *orf45*, show high homology to replication initiator proteins. The protein coded by *orf22* (367aa) shows absolute identity to the replication initiation protein RepA of plasmid pUT1 of *S. japonicum* pUT26S (Nagata *et al.* 2010). It contains a sequence that shows similarity with the well-defined RPA and Rep-3 domains. The proteins having these conserved domains have been shown to play a key role in replication initiation of plasmids (Wickner *et al.* 1991). As in plasmid pUT1, in the upstream of *repA*, an *oriV* sequence was identified with characteristic iterons, an AT-rich sequence, and a *dna*A box (Figure 2). Iterons play a key role both in initiation and regulation of replication in theta-replicating plasmids (del Solar *et al.* 1997; Nordstrom 1990). There is a significant similarity among the *oriV* sequences found in plasmids pPDL2, pUT1, and pPS10 (Figure 2). If the structural conservation of both *oriV* and RepA is taken into consideration, they qualify to have all the potential to replicate plasmid pPDL2 (Figure 2). To gain further evidence on their role in pPDL2 replication, we generated a mini-replicon by ligating the *repA oriV* region (18,815–20,914) to a tetracycline-resistant gene. When electroporated into *Sphingobium fuliginis* ATCC 27551, the resultant mini-replicon (pSFT2) gave tetracycline-resistant colonies, indicating the predicted *oriV* and *repA* contribution for the replication of pPDL2. Further, the generated mini-replicon remained in the cells even after growing them in the absence of antibiotic for several generations. Existence of the mini-replicon was also evident in plasmid preparations made from the transformed cells (data not shown). The RepA sequences of theta-replicating plasmids have been showed to follow a characteristic phylogenetic pattern and, to some extent, serve as signatures when assigning incompatibility groups to the plasmids (del Solar *et al.* 1997; Suzuki *et al.* 2010). Therefore, the RepA sequence of plasmid pPDL2 was aligned with the available RepA sequences in the GenBank (Suzuki *et al.* 2010). RepA of pPDL2 has more than 50% identity covering the complete sequence of RepA sequences of the IncP group of plasmids, pADP-1, pEST4011, and pUB1 (Suzuki *et al.* 2010). Such similarity is not seen with RepA sequences of other incompatibility groups of plasmids, suggesting that pPDL2 could be an IncP group of plasmid.

The protein coded by *orf24* of pPDL2 has 100% sequence coverage and identity with the partitioning protein ParA of plasmid pUT1 of *S. japonicum* UT26S. ParA, in association with ParB, plays a major role in plasmid segregation. However, no *parB* homolog is found in pPDL2. In the absence of *parB*, pPDL2 may depend on a ParB protein encoded either by the chromosome or by other indigenous plasmids present in *S. fuliginis* ATCC 27551. Interestingly, a well-defined plasmid addiction module was identified in pPDL2. The ORFs *orf29* and *orf30* code for proteins showing more than 95% identity to the RelB/RelE toxin/antitoxin system found in a variety of Gram-negative bacteria and archaea (Gotfredsen and Gerdes 1998; Gronlund and Gerdes 1999). The RelE and RelB pair of *E. coli* is well characterized. The 11kD RelE protein acts as toxin to bacterial cells, and a similarly sized *relB* product counteracts the toxic effect of RelE (Gotfredsen and Gerdes 1998; Gronlund and Gerdes 1999). Presence of partition protein ParA, along with a post-segregational killing mechanism (RelE/RelB), suggests faithful partitioning and maintenance of pPLD2 in daughter cells of *Sphingobium fuliginis* ATCC 27551.

### Plasmid pPDL2 is a mobilizable plasmid

**Figure 3:**
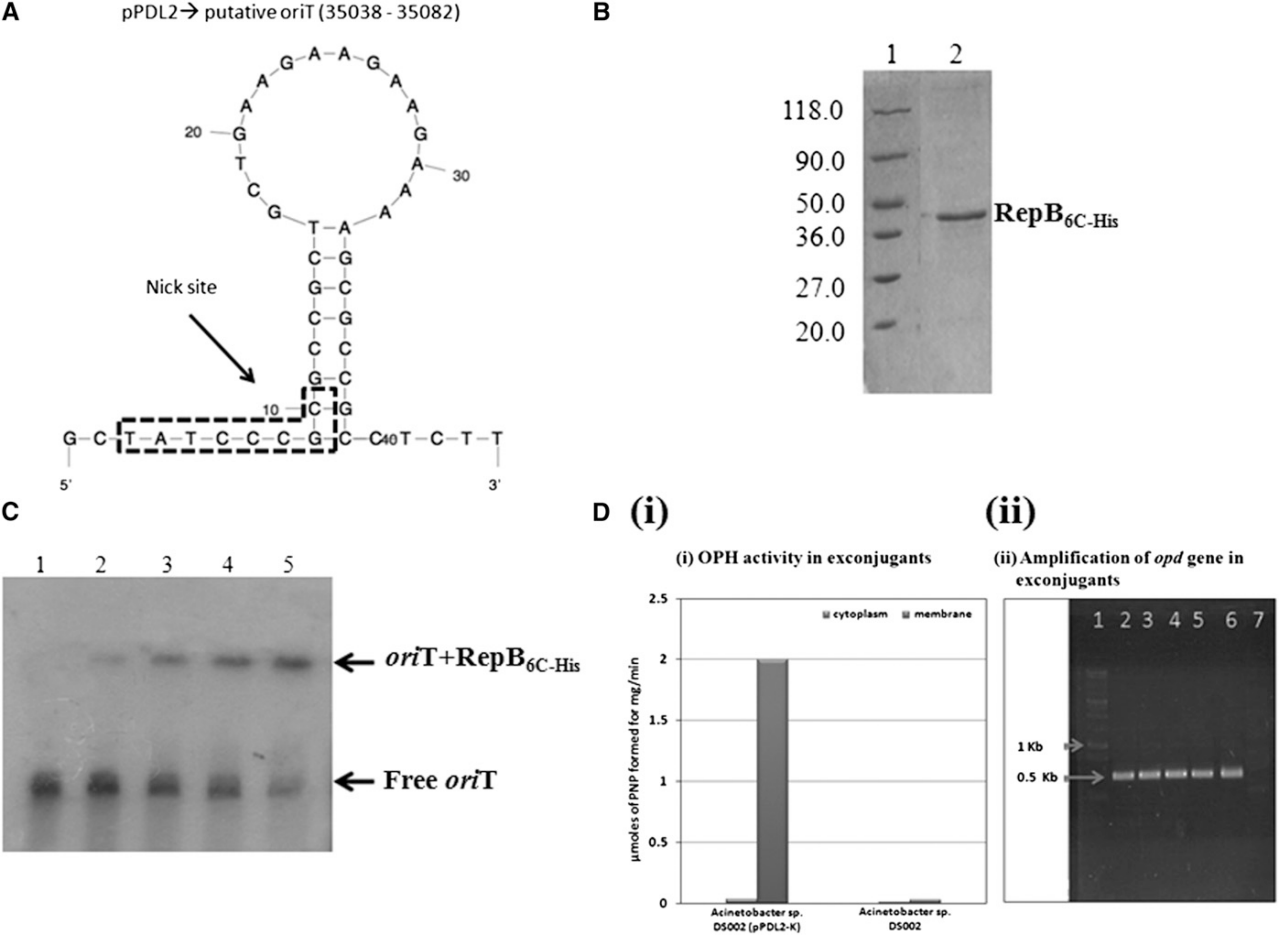
Mobilization of pPDL2. Panel A represents secondary structure of predicted *ori*T sequence. The nick site that matches perfectly with the nick site of plasmid pLB1 of *Sphingobium japonicum* is shown in a dotted box. Affinity purification of RepB_C-6His_ is shown in panel B. Panel C indicates the mobility shift assay done using radiolabeled *ori*T fragment and purified RepB_C-6His_. Lane 1 represents ^32^P labeled *ori*T without RepB_C-6His_. Lanes 2–5 represent labeled *ori*T incubated with increased concentrations [0.1 μg (2), 0.25 μg (3), 0.5 μg (4), and 1 μg (5)] of RepB_C-6His_. Arrows indicate either free *ori*T or *ori*T-RepB_C-6His_ complex. OPH activity (i) and amplification of *opd* gene (ii) in exconjugants are shown in panel D.

Since *oriT* is an essential element for mediating horizontal gene transfer we searched for a similar sequence in pPDL2. A number of functionally validated *oriT* sequences have been shown to contain typical secondary structures in a characteristic GC rich region (Lee and Grossman 2007) and such an *oriT*-like sequence (34823-35011) was identified in pPDL2. The predicted *oriT* has very high similarity to an *oriT* sequence of a self-transmissible plasmid pLB1 of *S. japonicum* UT26S (Miyazaki *et al.* 2006). If the location of *oriT* is taken into consideration, along with the presence of four tandem repeats immediately downstream, the predicted sequence has all features of a functional *oriT* (Figure 3A). Interestingly the putative *oriT* sequence is found upstream of an ORF (*orf45*) that codes for a protein showing high similarity (98%) to the plasmid-borne RepB sequences of *Sphingobiaceae* members (Bertalan *et al.* 2009).

The predicted RepB sequence includes a Rep-3 domain, involved in typical nicking-closing like activity (Dyda and Hickman 2003; Moscoso *et al.* 1995). Proteins having a Rep-3 domain also perform strand-transfer reactions generally seen during rolling circle (RC) replication (Moscoso *et al.* 1995; Ruiz-Maso *et al.* 2007). RC replication is also seen during horizontal transfer of indigenous plasmids. However, it is initiated from a well-defined *oriT* sequence (Pansegrau and Lanka 1991). Since we have identified both *oriT* and RepB in the sequence of pPDL2 we have attempted to validate their involvement in the lateral transfer of pPDL2. We initially assessed whether pPDL2 is a mobilizable plasmid by performing triparental mating experiments, involving *E. coli pir*-116 (pPDL2-K) as donor, with *Acinetobacter* sp. DS002 and *E. coli* (pRK2013) as recipient and helper strains respectively. Kanamycin-resistent exconjugants of *Acinetobacter* sp. DS002 have appeared on a selection plate at a frequency of 3.4x10^−5^ per recipient. As expected, all exconjugants carried the *opd* gene and showed OPH activity, properties not found in *Acinetobacter* sp. DS002 (Figure 3D). Mobility of plasmid pPDL2 was also observed when *E. coli* BL21 was used as recipient. Though pPDL2 is stably replicated in *Acinetobacter* sp. DS002, it failed to replicate in an *E. coli pir* negative background. Although kanamycin resistant *E. coli* colonies were found, they subsequently lost the plasmid. In certain colonies that retained the ability to grow on kanamycin we could not detect plasmid from these cells suggesting that either complete pPDL2-K or the region containing kanamycin gene has probably integrated into the genome.

To understand RepB and *oriT* interactions better we performed mobility shift assays by incubating the PCR amplicon carrying *oriT* with purified RepB_C-6His_ (Figure 3B). There was a RepB_C-6His_ dependent shift in the electrophoretic mobility of *oriT* (Figure 3C) and no shift in presence of a BSA control, indicating specific interactions between RepB_C-6His_ and *oriT*. Supporting this observation the intensity of shifted *oriT* band got proportionately reduced when increased concentrations of cold *oriT* was added to the reaction mixture (data not shown). To assess if RepB might function as a relaxase (Mob) the sequence of RepB was aligned with other relaxase sequences (Achtman 2011; Francia *et al.* 2004; Garcillan-Barcia *et al.* 2009; Guglielmini *et al.* 2011) and a weak homology was observed with relaxase of the MOB_F_ plasmid. The role of RepB in lateral transfer of pPDL2, therefore needs to be further investigated. Despite not detecting *tra* genes in the sequence of pPDL2, experiments were also performed to assess whether pPDL2 is self-transmissible but no exconjugants were observed from a biparental mating using *E. coli pir*-116 (pPDL2-K) as donor and *Acinetobacter* sp. DS002 as recipient. Hence, in summary, our data clearly indicate that pPDL2 is a mobilizable but not a self-transmissible plasmid.

### Integration module

**Figure 4:**
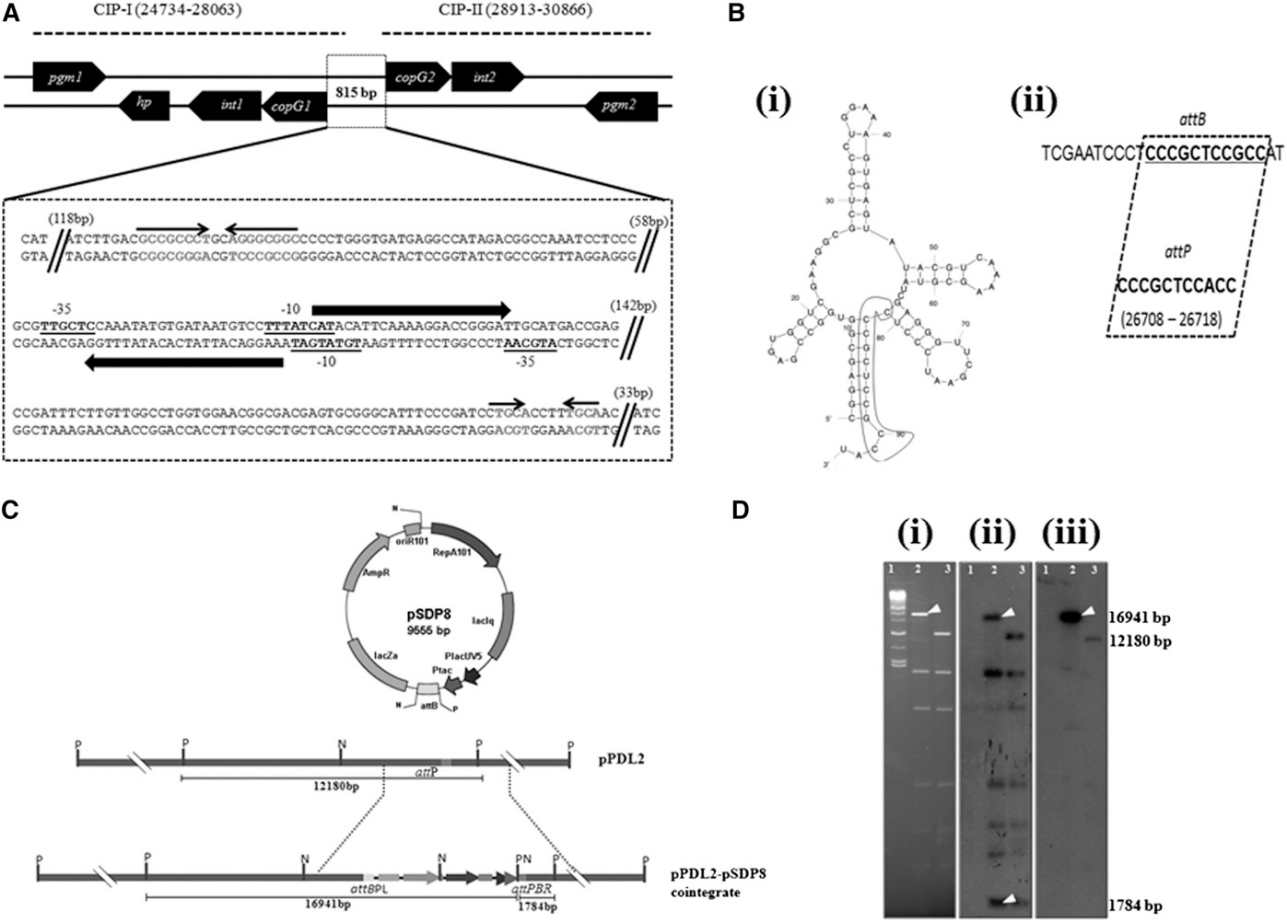
Site-specific recombination. Integration of artificial plasmid pSDP8 at the predicted *attP* site of pPDL2. Panel A represents physical map of integration module. The underlined sequences indicate putative promoter elements. The CopG binding sites are shown with inverted arrows. The transcription orientation of integration modules I and II are shown with solid arrows. The synthetic *attB* site created using sequence of *Sphingobium japonicum* UT26S and the similarities between predicted pPDL2 *attP* and *attB* sequences are shown in (i) and (ii) of panel B, respectively. The physical map of pSDP8 and site of integration of pSDP8 in pPDL2 is shown in panel C. The restriction profile of the cointegrate (i) and the corresponding southern blot developed using either labeled pPDL2 (ii) or pSDP8 (iii) are shown in panel D. The increase in size of 4.7 kb *Pst*I fragment due to integration of a 6.4 Kb plasmid pSD8 is shown with an arrow.

An interesting observation in the sequence of pPDL2 is the existence of an uniquely organized integrase module (nucleotide position 26697 to 30249). The module contains two units, CIP-I and CIP-II, each consisting of *copG*, *int* and *pgm* genes and having divergent transcription orientation (Figure 4A). The integrase of CIP-I is a 328 amino acid protein with strong identity (86%) to a plasmid pUT1-borne phage integrase of *S. japonicum* UT26 and 70% identity to a site-specific recombinase of *Pseudomonas syringae* DC 3000. The CopG/MetJ/Arc family regulatory protein (subsequently referred to as CopG) coded by pPDL2 shows highest similarity to the CopG protein coded by plasmid pUT1 of *S. japonicum* UT26S. The 131 residue protein shows homology to a number of other proteins belonging to CopG/MetJ/Arc family of transcriptional regulators that can act both as transcriptional repressors and activators (del Solar *et al.* 1995; del Solar and Espinosa 1992; Gomis-Ruth *et al.* 1998; He *et al.* 2002). In a well-characterized Streptococcal plasmid, pMV185, CopG has been shown to act as a repressor of *repB* by binding to a pseudosymmetirc region overlapping the -35 sequence of the σ^70^-dependent promoter, thereby preventing transcription of the *copG*, *repB* operon in plasmid pMV158 (Costa *et al.* 2001).

In pPDL2, the *copG* and *int* genes appear to be organized as one operon in both CIP-I and CIP-II units. A potential σ^70^-dependent promoter was only seen upstream of each of the *copG* sequences, and the stop codon of *copG* overlaps with the start codon of the *int* genes suggesting that these two genes are cotranscriptional. Due to the existence of opposite transcriptional orientation, an overlap of 5bp is seen between the putative *copG1* and *copG2* promoters (Figure 4A). As CopG1 proteins are known to act as transcription repressors, the intergenic region found between the *copG1*, *int1* and *copG2*, *int2* operons was analyzed to identify *cis*-elements that can serve as target sites for CopG proteins (Figure 4A). In studies conducted by del Solar and his associates, CopG is shown to bind to a pseudosymmetric *cis*-element present overlapping -35 region (del Solar *et al.* 2002). Inverted repeats are also shown to be putative binding sites of CopG in *Sulfolobus neozealandicus* (Greve *et al.* 2005). Interestingly, in both CIP-I and CIP-II units, typical CopG binding sites were predicted upstream of the start codon of *copG* and putative promoter sequence (Figure 4A). Transcriptional organization of *copG* and *int* genes and the existence of such CopG binding sites, clearly indicates involvement of CopG in regulation of expression of the integrase in *S. fuliginis*ATCC 27551.

In addition to these two transcriptionally coupled integrase and *copG* genes an additional ORF that codes for a protein showing identity (98%) to phosphoglycerate mutase of *S. japonicum* UT26S is present at the 3′ end of the *int* gene in each of the two units of the integrase modules (Figure 4A). Though, the phosphoglycerate mutase gene in CIP-I module is found 1.1 Kb away from the stop codon of integrase, in CIP-II, it is found immediately downstream of transcription terminator sequence of *int2* gene. This 206 residue protein shows high identity (98%) to phosphoglycerate mutase encoded on plasmid pUT1 of *S. japonicum* UT26S. Phosphoglycerate mutases (PGMs) are very well characterized group of enzymes found both in prokaryotes and eukaryotes. PGMs are basically transferases involved in transfer of phosphate group (Parkinson and Kofoid 1992). Conversion of glyceraldehyde 3-phosphate to glyceraldehyde 2-phosphate, by transfer of phosphates from the 3^rd^ position to the 2^nd^ position, is the classical biochemical reaction catalyzed by this group of enzymes. However, PGMs containing an HP-PGM-like domain are also known to be involved in signal transduction process (Matsubara and Mizuno 2000). The histidine present in the catalytic site of this group of enzymes undergoes phosphorylation during the signal relay process (Parkinson and Kofoid 1992). The strong link between PGM, CopG and integrase suggests a related function of these proteins, and a similar organization is also seen in *S. japonicum* UT26S plasmids pCHQ1 (NCBI Reference no: NC014007) and pUT1 (NCBI Reference no: NC014005), and *Sphingomonas wittichi* RW1 (pSWIT01) adding strength to the proposed hypothesis.

To assess whether the CIP-I and CIP-II units were generated by gene duplication we compared the sequences of these two units. If they are products of gene duplication there should have been absolute identity between the proteins coded by these two units of the integrase module. However there is only 67% identity between these two proteins. The N-terminal region of CopG2 encoded by CIP-II unit was found to be much longer. In addition to the size difference, there was no sequence conservation at the C-terminus of the protein. Nevertheless, the central regions of CopG1 and CopG2 are almost identical. Such diversity in the primary sequence suggests a divergent origin of CopG1 and CopG2. A similar situation was seen when *int1* and *pgm1* were aligned with *int2* and *pgm2*. Between *int1* and *int2* only 80% identity was observed although this identity continued throughout the sequence. The percent identity between PGM1 and PGM2 is high (91%), the difference only being seen at the C-terminus and N-terminus. Considering the diversity in the primary sequence of the proteins coded by CIP-I and CIP-II of the integrase module, the plasmid pPDL2 is proposed to have acquired the CIP-I and CIP-II integrase modules from independent sources, possibly through a unique recombination process.

### Plasmid pPDL2 is an integrative mobilizable element

Having identified an integrase module, we performed experiments to determine whether site-specific integration of pPDL2 takes place at an artificially created *att*B site cloned in temperature-sensitive plasmid pDSP8. Initially we performed bioinformatic studies to identify *attP* sites on the plasmid pPDL2. While perfoming these predictions, we took tRNA genes from the genome sequence of *S. japonicum* UT26S, whose total genome sequence is known (Nagata *et al.* 2010). The reasons for taking tRNA genes from *S. japonicum* are that parts of pPDL2 show considerable homology with its indigenous plasmid pUT1 and that it encodes an integrase module very similar to the one present in pPDL2. These tRNA gene sequences were then blasted with the total sequence of pPDL2 to obtain a library of identical sequences with a minimum length of 10 bp. Among these sequences, the ones found in intergenic regions were predicted to be potential *attP* sites. The seryl tRNA gene of *S. japonicum* has sequence identity with the predicted *attP* at its 3′ end and served as an *att*B sequence [Figure 4B, part (i)]. *E. coli pir*-116 strains carrying pPDL2-K and pSDP8 were grown independently for 12 hr at 30° and 37°, and then plated on kanamycin and ampicillin plates. A lawn of cells were seen on plates seeded with the 30°-grown culture, whereas only a few colonies appeared in plates seeded with the 37°-grown culture. In the absence of *attB*, no colonies appeared, suggesting that the generated colonies were due to occurrence of site-specific recombination between *attB* and *attP* sites [Figure 4B, part (ii)]. The restriction profile of plamids isolated from randomly picked colonies showed integration of pSDP8 in a 12.0 kb *PstI* fragment of pPDL2 (Figure 4, C and D), and sequence analysis revealed *att*PBL and *att*BPR sequences, suggesting that the *att*P site predicted at the 3′ end of *int1* served as an *attP* site in pPDL2.

### Degradation module

In our previous studies, we analyzed the 7 kb DNA region of pPDL2 that shows sequence identity with the pCMS1-borne *opd* cluster from *B. diminuta*, and we identified a transposon-like organization of the *opd* gene cluster in *S. fuliginis* ATCC 27551 (Siddavattam *et al.* 2003). Such structural organization and the existence of identical *opd* clusters in bacterial strains representing diverse taxonomic groups suggested horizontal transfer of the *opd* cluster through transposition (Figure S1). However, in our previous studies, we were unable to detect any transposition events, and we concluded that it is a defective transposon due to the existence of a truncated *tnpA* gene in the Tn*3* element identified at the flanking end of the *opd* cluster.

**Figure 5:**
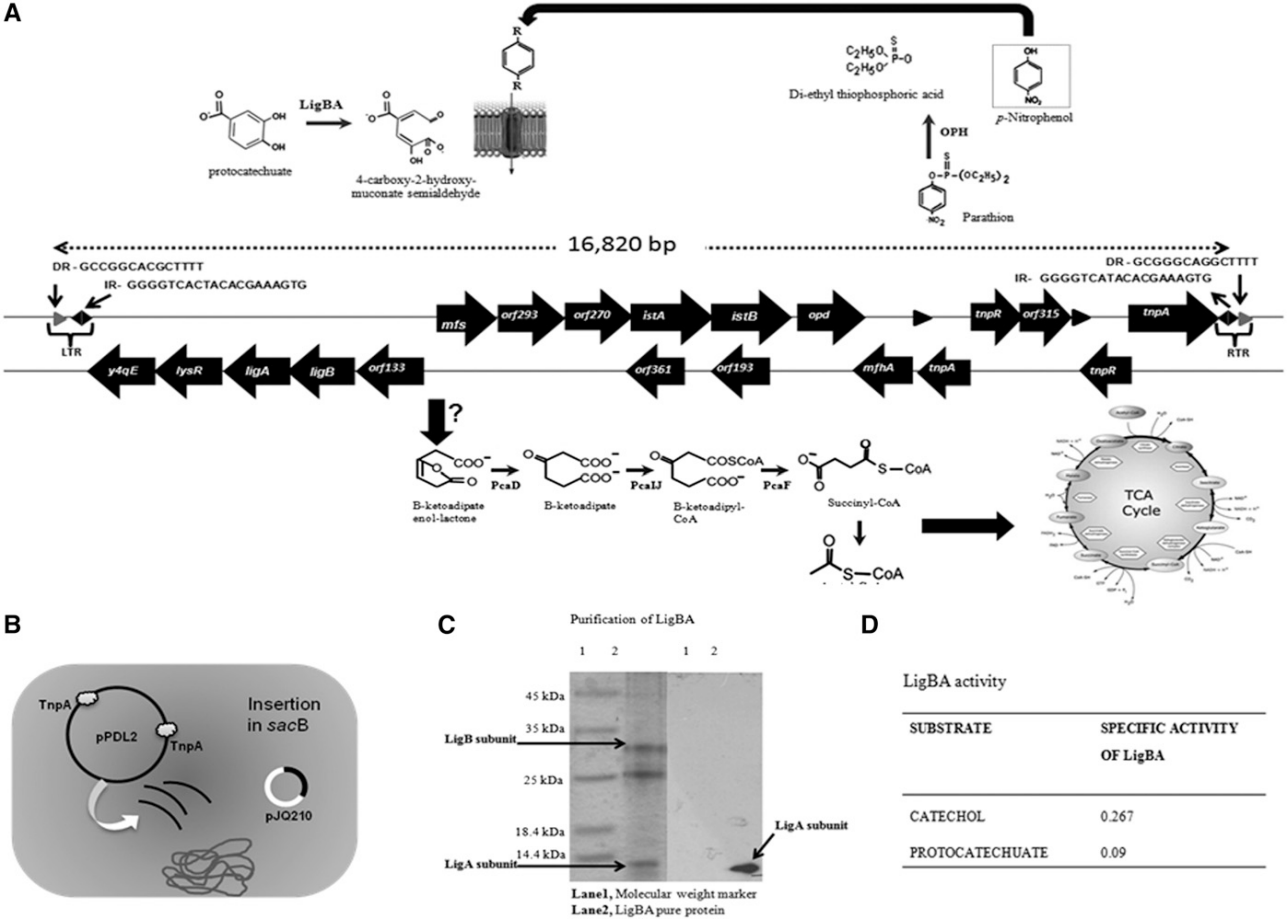
Transposition assay. Panel A indicates the physical map of the degradative module. The Tn*3*-specific terminal repeats found upstream and downstream of transposable element Tn*3* and *y4qE* are shown with arrows. The reactions catalyzed by OPH, LigBA are shown. The transposon assay performed to assess transposition of the degradative module is shown in panel B. Panel C indicates affinity purification of LigBA and the corresponding Western blot using anti-His antibody. The dioxygenase activity of purified dioxygenase (LigBA) with various aromatic compounds is shown in panel D.

The full sequence of pPDL2 shows the existence of second copy of Tn*3* immediately downstream of the defective Tn*3* element, and so we have revisited the question of whether the *opd* cluster of pPDL2 functions as an active transposon. Bioinformatic analysis identified Tn*3*-specific terminal repeats (Siguier *et al.* 2006) in pPDL2: two at the terminal ends of Tn*3* and a third at the upstream region of transposon *y4qE* (Figure 5A). The genetic map of the DNA region between *y4qE* and Tn*3* includes the previously reported *opd* gene clusters (Siddavattam *et al.* 2003), transposons Tn*3* and *y4qE*, and the *ligBA* operon coding for the protocatechuate-4,5-dioxygenase alpha and beta subunits, along with a gene (*orf1*) coding for a LysR-type transcription regulator. The *ligBA* operon codes for protocatechuate-4,5-dioxygenase alpha and beta subunits showing 83% identity to the similar proteins of *Xanthomonas campestris pv. campestris* str. ATCC 33913 (NCBI reference no. NP_636196.1). While evaluating the functional status of *lig* operon, an expression plasmid (pSDP9) was constructed by placing it under the control of vector-specific transcriptional and translational signals. The resting cells prepared from induced cultures of *E. coli* BL21 (pDSP9) successfully generated ring-cleavage products from proctocatechuate, catechol, and benzenetriol indicating the operon codes for a functional dioxygenase. As the cloning strategy translationally fuses *ligA* with vector sequences coding for a His-tag, the *E.,coli* BL21 (pDSP9) cells encode a LigA_C-6His_ protein. Affinity purification yielded both LigA_C-6His_ and LigB in approximately equimolar concentration, revealing that these two functionally related proteins form a complex (Figure 5A). Supporting the resting cell assay, the affinity-purified dioxygenase also generated ring-cleavage products from both protocatechuate and catechols (Figure 5D). In addition to LigB and LigA_C-6His_ proteins, an additional band with a size of 25 kD was enriched during affinity purification (Figure 5C). It is not clear whether this 25 KDa protein is copurified due to specific interactions with Lig proteins. Protocatechuate-4,5-dioxygenase is the key enzyme in the benzoate (hydroxylation pathway) and 2,4-dichlorobenzoate degradation pathways (Adriaens *et al.* 1989). In addition to these two compounds, many aromatic compounds such as vanillate, isovanillate, phthalates, and benzoate derivatives are shown to be channelled through the protocatechuate degradation pathway (Providenti *et al.* 2006). When OP insecticides, like methyl parathion and parathion, are hydrolyzed by OPH, aromatic compounds such as *p*-nitroarophenols are generated (Sethunathan and Yoshida 1973). These compounds are found to be more toxic to the microflora than the parent compounds (US Environmental Protection Agency 2007). The existence of a ring-cleavage dioxygenase as part of the *opd* gene cluster allows channeling of these aromatic byproducts of OP insecticides into the TCA cycle, contributing to their complete mineralization.

In addition to the *lig* operon, *lysR* and two ORFs showing homology to transporter proteins belonging to the major facilitator superfamily were identified in the upstream and downstream region of the *lig* operon (Figure 5A). Transporters belonging to the major facilitator superfamily proteins are known to be involved in transport of aromatic proteins (Pao *et al.* 1998). Hence, in summary, if the sequence found between the Tn*3*-specific terminal repeats is a transposable unit, it appears to have all necessary information for mineralization of OP insecticides like parathion and methyl parathion.

### The *opd* cluster of pPDL2 is an active transposon

The transposition assay that we described in our previous study was repeated by replacing pMS3 with the pPDL2-K derivative pPDL2-KT in which *opd* is replaced with *opd*::*tet* and hence the event of transposition can be monitored by the tetracycline-resistant phenotype. Plasmid pPDL2-KT requires *pir* protein for its replication and hence replicates only in *E. coli pir*-116 cells. When pPDL2-KT was electroporated into *E. coli* (pJQ210) cells, we found colonies resistant to sucrose and tetacycline, indicating disruption of the *sacB* gene of pJQ210 due to transposition of *opd*::*tet*. Further analysis of plasmids isolated from the sucrose/gentamycin/tetracyclin resistant colonies gave a typical restriction profile suggesting existence of *opd* cluster in the *sacB* region of pJQ210. The junctions of the *opd* cluster were then amplified using *sacB*-specific primers and primers designed for the region upstream of the predicted terminal repeats. The sequence of the PCR amplicons confirmed the existence of Tn*3*-specific terminal repeats, indicating that the *opd* cluster of pPDL2 is an active transposon (Figure 5B).

## DISCUSSION

Horizontal gene transfer in bacteria is an important driving force that plays a critical role in generating genetic variation among bacteria and contributes to shaping of their genomes (Hacker and Carniel 2001; Jain *et al.* 1999, 2003; Preston *et al.* 1998; Skippington and Ragan 2011). DNA acquisition by bacteria through various mobile genetic elements, such as plasmids, bacteriophages, IS elements, transposons, integrons, and conjugative transposons, is an established phenomenon (Dobrindt *et al.* 2004; Garcillan-Barcia *et al.* 2011; Wiedenbeck and Cohan 2011). Sequences associated with mobile genetic elements differ from the host genome with respect to their GC composition and GC skew, serving as unique makers to assess lateral acquisition. Such delineation represents an island-like existence for mobile genetic elements in the genome of the host (Juhas *et al.* 2009). Depending on the nature of the genetic information present on these mobile genetic elements, they are designated as pathogenic islands (Dobrindt *et al.* 2004; Schmidt and Hensel 2004), resistant islands (Larbig *et al.* 2002), or catabolic islands (Gaillard *et al.* 2006). Involvement of plasmids and catabolic transposons in lateral transfer of degradative genes is a recognized phenomenon (Topp *et al.* 2000; Tsuda *et al.* 1999; van der Meer *et al.* 2001). Recently genes required for 3-chlorobenzoate degradation have been shown to be part of an integrative conjugative element (ICE). This ICE element, found downstream of a glycyl tRNA gene, contains all the necessary genes for mineralization of 3-chlorobenzoate along with an *int* gene coding for a site-specific integrase and attachment sites (Gaillard *et al.* 2006).

Plasmid pPDL2 is unique in number of ways. It contains a well-defined replication module with an *oriV* sequence and a *repA* gene to support its replication. A mini-replicon generated by ligating the *oriV*, *repA* region of pPDL2 with an insertionally inactivated *opd* (*opd*::*tet*) gene successfully replicated in *Acinetobacter* sp. DS002, suggesting its involvement in replication of pPDL2. The pPDL2 also has an integrase module, consisting of *copG*, *int*, and *pgm* genes (Figure 4A). The organization of the integrase module is similar to the one present in plasmid pUT1 of *S. japonicum* UT26S, although pUT1 has a single integrase module, whereas pPDL2 has two modules. The data presented in the present study clearly show that pPDL2 can successfully integrate if a suitable *att*B site is available in the host chromosomal DNA. Plasmid pPDL2 contains an *oriT* sequence that is identical to the *oriT* sequence of pLB1 of *S. japonicum* UT26. In support of this observation, the pPDL2 derivative pPDL2-K was mobilized into *Acinetobacter* and *E. coli* cells in the presence of a helper strain *E. coli* HB101 (pRK2013). The data obtained in the present study clearly show formation of cointegrates between pPDL2-K and pSDP8 due to site-specific recombination. If a suitable *att*B site is present, such an event is possible between pPDL2-K and the *E. coli* chromosome. If this property of pPDL2 is taken together with its ability to mediate its own mobilization, designating pPDL2 as an IME appears to be logical. In general, lateral mobility of *Sphingobium* plasmids is only seen among *Sphingomonadaceae* members (Basta *et al.* 2004). However, the self-transmissible *Sphingobium* plasmid pLB1 derivative pLB1-K has been successfully transmitted from *S. japonicum* to *Sinorhizobium meliloti* and *Mesorhizobium loti* (Miyazaki *et al.* 2006). Like pLB1, plasmid pPDL2 derivative pPDL2-K can be successfully mobilized from *E. coli* to *Acinetobacter* that is not a member of the *Sphingomonadaceae*. This property of pPDL2 facilitates lateral transfer of the *opd* cluster among a wide range of bacterial strains. When such lateral transfer takes place into a host where pPDL2 replication is not supported, it can integrate into the genome of the recipient cells due to the existence of an integrase module. This additional feature appears to ensure retention of genetic information evolved for degradation of organophosphates, even in a host that does not permit replication of pPDL2.

Furthermore, pPDL2 contains a catabolic transposon that contains information for hydrolyzing a structurally diverse group of organophosphates and then for degrading aromatic compounds generated due to OPH-mediated hydrolytic cleavage of OP insecticides. As shown in Figure 5A, the entire degradative module appears to be organized as a complex transposon having transposons *y4qE* and Tn*3* at the flanking ends of degradative modules. Tn*3*-specific terminal repeats were found upstream and downstream of *y4qE* and Tn*3*, and consistent with this sequence information, we demonstrated a transposition event that inserted the entire degradative module into the *sacB* gene of a reporter plasmid. This event suggests the possibility of spreading *opd* as part of a large catabolic transposon. Surprisingly, most of the genetic information found on 37,317 kb pPDL2 appears to be related either to lateral gene transfer or degradation of organophosphates. It appears that the plasmid is tailor-made through a number of recombination and transposition events for spreading *opd* information among soil bacteria. The presence of identical *opd* regions on plasmids pCMS1 and pPDL2 and the existence of an active transposable element with *opdA* is in itself evidence for acquisition of *opd* through lateral gene transfer (Mulbry *et al.* 1987; Horne *et al.* 2002, 2003; Pandeeti *et al.* 2011). In the *Sphingobiaceae* family, only two members, *S. fuliginis* ATCC 27551 and *Sphingomonas* sp. JK1, have *opd* genes (NCBI reference no. ACD85809). The existence organophosphates in the soil might have favored the *opd*-containing bacteria to acquire DNA that encodes transporters and aromatic degradation genes as it allows the complete utilization of organophosphates.

Considering the experimental data presented in this study, we have searched genome sequences of *Sphingobiaceae* members to determine whether sequences that match pPDL2 exist in other genomes. Synteny maps were drawn to show the extent of similarity between pPDL2 and genome sequences of Sphingobiaceae members (*S. japonicum* and *S. wittichii*). Plasmid pUT1 of *S. japonicum* has organizational similarity with pPDL2 in the region containing replication and integrase genes (see Figure S2). If synteny maps are taken into consideration along with GC content and GC skew, it clearly suggests that plasmid pPDL2 evolved by a number of transposition and rare recombination events to integrate DNA from different sources. Although pPDL2 has all the necessary features for spreading *opd* information, due to the lack of *tra* genes, it depends on helper plasmids for achieving lateral transfer. However, as *Sphingobium fuliginis* ATCC 27551 has three more plasmids, complementation of such a function by these plasmids cannot be ruled out.

## Supplementary Material

Supporting Information
