# Supplementary material for: Multiple Mechanisms Contribute to Lateral Transfer of an Organophosphate Degradation (opd) Island in Sphingobium fuliginis ATCC 27551
Source: G3 (Bethesda). 2012 Dec 1;2(12):1541–54. doi: 10.1534/g3.112.004051 (PMC3516476; doi:10.1534/g3.112.004051)
Supplement: Supporting Information [file supp_2_12_1541__index.html]

Supporting Information 

# Multiple Mechanisms Contribute to Lateral Transfer of an Organophosphate Degradation (*opd*) Island in *Sphingobium fuliginis* ATCC 27551

## Supporting Information for Pandeeti *et al.*, 2012

**Files in this Data Supplement:**

- Supporting Information - Figures S1-S2 and Tables S1-S2 (PDF, 1 MB)
- Figure S1 - Geographical distribution of *opd* genes (PDF, 263 KB)
- Figure S2 - Genetic map of *oriV* and *int* regions (PDF, 876 KB)
- Table S1 - List of primers used in this study (PDF, 63 KB)
- Table S2 - Genome Inventory of pPDL2 (PDF, 84 KB)
