# Supplementary material for: Multiple Mechanisms Contribute to Lateral Transfer of an Organophosphate Degradation (opd) Island in Sphingobium fuliginis ATCC 27551
Source: G3 (Bethesda). 2012 Dec 1;2(12):1541–54. doi: 10.1534/g3.112.004051 (PMC3516476; doi:10.1534/g3.112.004051)
Supplement: Supporting Information [file supp_2.12.1541_004051SI.pdf]

A

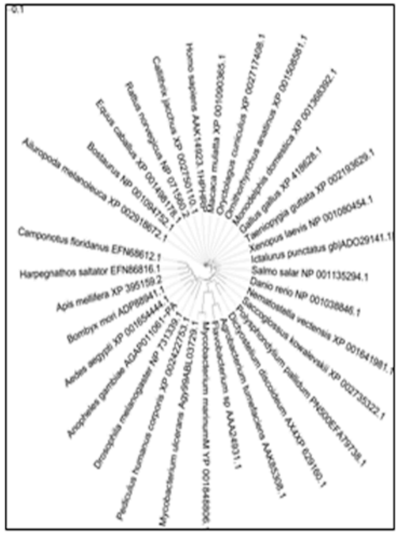

B

Bacteria strains	Location of *opd/opd*A gene	Geographical distribution	Phylogenetic Distribution (Class)	Accession Number of the sequence
Brevundimonas diminuta strain MG	Plasmid pCMS1	USA (Texas)	α-proteobacteria	HQ839670.1
Flavobacterium(Sphingomonas fuliginis) sp. ATCC 27551	Plasmid pPDL2	Philippines (IRRI)	α-proteobacteria	AJ421424.1
Flavobacterium balustinum	Plasmid pBC9	India (Anantapur)	Flavobacteria	AJ426431.1
Sphingomonas sp. JK1	Unknown	India (Mumbai)	α-proteobacteria	EU709764.1
Agrobacterium tumefaciens	Chromosomal	Australia	α-proteobacteria	AY043245.2

Figure S1 Geographical distribution of *opd* genes

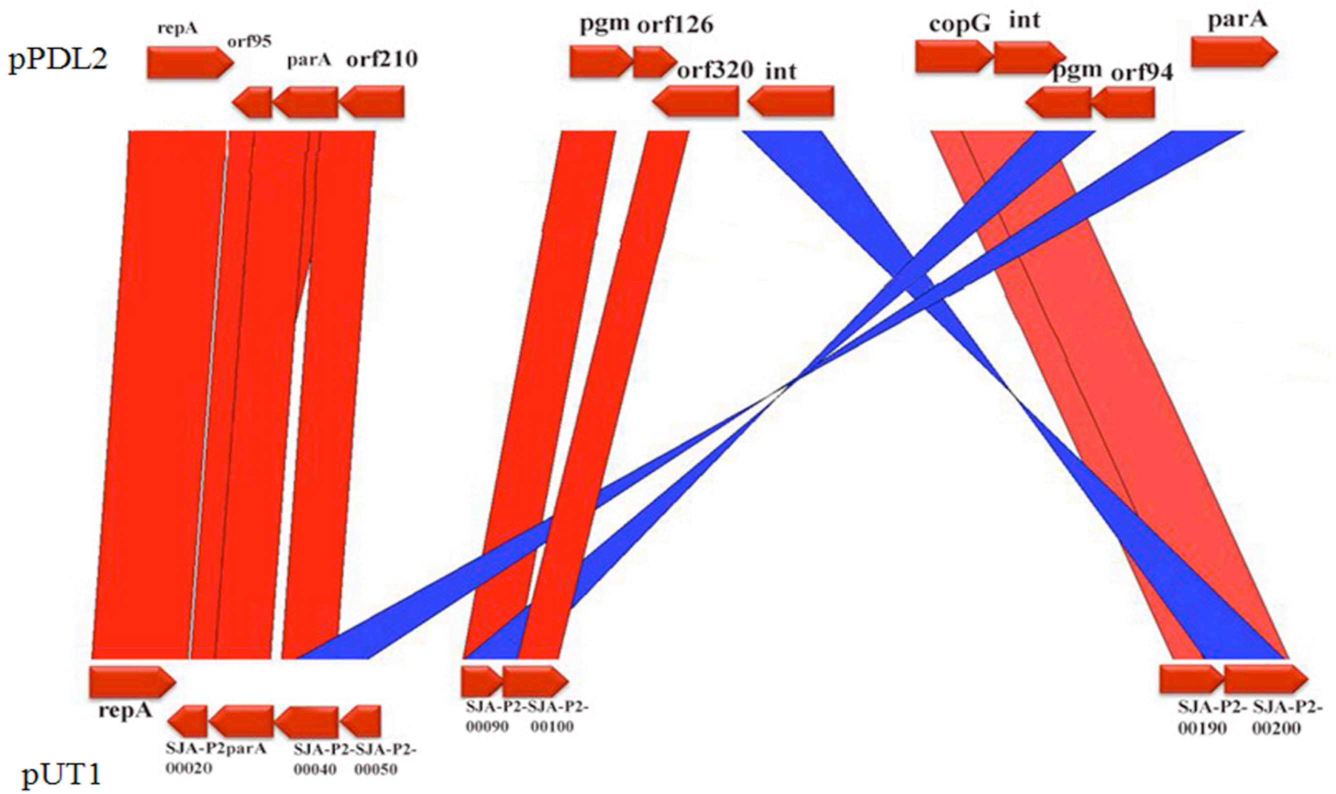

Figure S2 Genetic map of *oriV* and *int* regions

Table S1 List of primers used in this study

Name of the primers	Sequences	Description
M13F	TGTAAAACGACGGCCAGT(F*)	Universal sequencing primers
M13R	GATAACAATTTCACACAGGA(R*)	
DSF0001	CGGAGCGGTGGCCGAGTGGTCGAAGGCGCTCGCCTGGAAAGTGAGTATACGTCAAAAGCG(F*)	Overlapping primers used to synthesize seryl *tRNA* gene with *att*B at 3' end
DSF0002	ATGGCGGAGCGGGAGGGATTCGAACCCTCGATACGCTTTTGACGTATACTCACTTTCCAG(R*)	
DSF0003	GAGAATTCCGGAGCGGTGGCCGAGTGGT(F*)	Primers used to amplify *att*B sequence
DSF0004	GAGAATTCATGGCGGAGCGGGAGGGATT(R*)	
DSF0005	TCTTGAGATATCACTGATAGATACAAGAGC(F*)	Primers used to amplify *bla*, *oriR101* and *repA101*^{ts} sequences from pKD46
DSF0006	CAATAACCCGGGTAAATGCTTCAATA(R*)	
DSF0007	CGCGAGCATCAGGCTTGG(F*)	Primers used for *att*PBL amplification from cointegrate
M13R	GATAACAATTTCACACAGGA(R*)	
DSF0008	GGGCATGCGGTCGTCATCCTTGTGCAT(R*)	Primers used for *att*BPR amplification from cointegrate

M13F	TGTAAAACGACGGCCAGT(F*)	
DSF0009<br>DSF0010	GCTGAGGTGGATCCATGAATCATGCAAC(F*)<br>AGATAGGTCTCGAGTGCTAGGCAGGCGCAGCG(R*)	Primers used to amplify *repB*
DSF0011<br>DSF0012	GGCTTGCTGCAGGCGTGAGCACACCTA(F*)<br>GGCTACTGCAGGCGTATACAGCTATAC(R*)	Primers used to amplify *oriV* and *repA* of pPDL2
DSF0013<br>DSF0014	CCGGACCATATGACATGGCCGCTGCC(F*)<br>GCGGGAAGCTTCAGTTCAGGTGGCG(R*)	Primers used to amplify *ligA* and *ligB*
DSF0015<br>DSF0016	CTGGCAGGAGGCGCAACTCA(F*)<br>CAAGGATGCTGTCTTTGACAACAGATG(R*)	Primers used to amplify *sac*B
DSF0017<br>DSF0018	CTGACAATCGAGGAGCACTACAC(F*)<br>CTTAGTTGCAGAAATAGGCGACCTT(R*)	Primers used for amplification of right and left ends of predicted Tn3 transposon

* F= Forward primer/ R= Reverse Primer

Table S2 Genome Inventory of pPDL2

ORF	Feature	Position*	Determined or estimated function	% Amino acid identity	Source	Identification number of closest Relative
orf1	*lysR*	46-996’	LysR family transcriptional regulator	44(136/310)	*Xanthomonas campestris pv. campestris* str. ATCC 33913	NP_636197.1
orf2	*ligA*	1055-1423’	Protocatechuate 4,5-dioxygenase alpha chain	69(81/117)	*XXanthomonas campestris pv. vesicatoria* str. 85-10	YP_362645.1
orf3	*ligB*	1427-2308’	Protocatechuate 4,5-dioxygenase beta chain	78(221/282)	*Xanthomonas fuscans subsp. aurantifolii* str. ICPB 11122	ZP_06704385.1
orf4	*orf133*	2375-2776’	Hypothetical protein	46(64/110)	*Sphingobium* sp. SYK-6	YP_004835142.1
orf5	*orf241*	2769-3494’	Alpha/beta hydrolase fold protein	48(112/231)	*Rhodobacterales bacterium* HTCC2083	ZP_05073542.1
orf6	*Mfs*	3668-4567	Permeases of the major facilitator superfamily	71(177/248)	*Asticcacaulis excentricus* CB 48	YP_004088206.1
orf7	*Mfs*	4604-5122	Major facilitator superfamily MFS_1	73(125/172)	*Asticcacaulis excentricus* CB 48	YP_004088206.1
orf8	*Bla*	5310-6191	Beta-lactamase domain-containing protein	49(141/290)	*Rhodococcus pyridinivorans* AK37	ZP_09310177.1
orf9	*orf270*	6221-7033	Hypothetical protein Swit_1907	42(102/241)	*Sphingomonas wittichii* RW1	YP_001262405.1

orf10	*istA*	7242-8711	Transposase	70(336/479)	*Methylocystis* sp. ATCC 49242	ZP_08071202.1
orf11	*orf361*	7392-8477'	Orf361	31(64/204)	*Roseobacter* sp. GAI101	ZP_05102673.1
orf12	*orf193*	8654-9235'	Orf193	21(35/163)	*Legionella pneumophila* str. Paris	YP_123784.1
orf13	*istB*	8723-9547	Transposition helper protein	85(215/252)	*Methylocystis* sp. ATCC 49242	ZP_08072130.1
orf14	*opd*	9814-0911	Parathion hydrolase	100(365/365)		AER10490.1
orf15	*mfhA*	10942-11673'	*Meta*-fission product hydrolase	100(218/218)	*Brevundimonas diminuta*	AER10491.1
------	------	11600-11619	Tn3 repeat			
orf16	*tnpA*	11670-13301'	Transposase	95(322/338)	*Sphingomonas* sp. KA1	YP_717957.1
orf17	*tnpR*	13442-14011	Resolvase	100(189/189)	*Novosphingobium pentaromativorans* US6-1	ZP_09195142.1
orf18	*orf314*	14012-14959	Hypothetical protein	95(281/296)	*Novosphingobium pentaromativorans* US6-1	ZP_09195143.1
------	------	14838-14857	Tn3 repeat			
orf19	*tnpR*	14871-15545'	Resolvase	100(224/224)	*Sphingobium japonicum* UT26DB	YP_740317.1

orf20	*tnpA*	15604-18561	Transposase	99(984/985)	*Sphingobium japonicum* UT26DB	YP_740316.1
------	------	18599-18618	Tn3 repeat			
orf21	*orf183*	18620-19171	Hypothetical protein	100(183/183)	*Novosphingobium pentaromativorans* US6-1	ZP_09195143.1
orf22	*repA*	19777-20880	Replication protein	100(367/367)	*Sphingobium japonicum* UT26S	YP_003543403.1
orf23	*orf95*	20909-21196’	Hypothetical protein	100(92/92)	*Sphingobium japonicum* UT26S	YP_003543404.1
orf24	*parA*	21193-21828’	Partitioning protein	100(211/211)	*Sphingobium japonicum* UT26S	YP_003543405.1
orf25	*orf210*	21950-22582’	Hypothetical protein	96(201/210)	*Sphingobium japonicum* UT26S	YP_003543406.1
orf26	*orf73*	22963-23184’	Hypothetical protein	53(23/43)	*Sinorhizobium meliloti* AK83	YP_004557067.1
orf27	*orf127*	23657-24040	Hypothetical protein	26(23/89)	*Cenarchaeum symbiosum* A	YP_875497.1
orf28	*relB*	24190-24456	RelB antitoxin; DNA-damage-inducible protein J	98(86/88)	*Sphingobium japonicum* UT26S	YP_003547005.1
orf29	*relE*	24443-24724	RelE/StbE family addiction	96(89/93)	*Sphingomonas wittichii* RW1	YP_001259995.1

			module antitoxin; YafQ toxin protein			
orf30	*pgm*	24734-25354	Phosphoglycerate mutase family protein	96(197/205)	*Sphingobium japonicum* UT26S	YP_003543412.1
orf31	*orf126*	25354-25734	Hypothetical protein	38(27/72)	*Yersinia pestis* FV-1	ZP_02335234.1
orf32	*orf320*	25731-26693’	Hypothetical protein	99(156/158)	*Sphingobium japonicum* UT26S	YP_003543413.1
orf33	*Int*	26697-27671’	Phage integrase family protein	90(291/324)	*Sphingobium japonicum* UT26S	YP_003547002.1
-------	*attP*	26708-26718	Putative attachment site			
orf34	*copG*	27668-28063’	Putative transcriptional regulator CopG/Arc/MetJ family	89(116/131)	*Sphingobium japonicum* UT26S	YP_003547001.1
orf35	*orf124*	28115-28489’	Hypothetical protein	68(69/101)	*Sphingobium japonicum* UT26S	YP_003543420.1
orf36	*orf121*	28513-28878	Hypothetical protein	89(102/114)	*Sphingobium japonicum* UT26S	YP_003547000.1
orf37	*copG*	28913-29284	Putative transcriptional regulator CopG/Arc/MetJ family	70(90/128)	*Sphingobium japonicum* UT26S	YP_003547001.1
orf38	*Int*	29263-30249	Phage integrase family protein	95(305/322)	*Sphingobium japonicum* UT26S	YP_003547002.1
orf39	*pgm*	30246-30866’	Phosphoglycerate mutase family protein	90(185/205)	*Sphingobium japonicum* UT26S	YP_003543412.1

orf40	*orf94*	30873-31157’	Hypothetical protein	72(62/86)	*Methylosinus trichosporium* OB3b	ZP_06890525.1
orf41	*pilT*	31154-31573’	PilT domain-containing protein	60(84/139)	*Candidatus Glomeribacter gigasporarum* BEG34	ZP_08885245.1
orf42		32080-32715	Hypothetical protein	91(192/211)	*Sphingobium japonicum* UT26S	YP_003543406.1
orf43	*parA*	32796-33431	Chromosome partitioning protein	93(196/211)	*Sphingomonas* sp. S17	ZP_08389807.1
orf44		33451-33723	Hypothetical protein	83(76/92)	*Sphingomonas* sp. S17	ZP_08389792.1
orf45	*repB*	33795-34706’	Replication initiation protein	83(173/209)	*Gluconacetobacter diazotrophicus* PAl 5	YP_002278340.1
------	*oriT*	35038-35082	Putative oriT region			
------	------	35272-35291’	Tn3 repeat			
orf46	*Tnp*	35597-36700’	Transposase	58(211/362)	*Roseibium* sp. TrichSKD4	ZP_07662592.1

* = ‘ denotes that the existence of gene is on complementary strand.
