# Supplementary material for: Multiple Mechanisms Contribute to Lateral Transfer of an Organophosphate Degradation (opd) Island in Sphingobium fuliginis ATCC 27551
Source: G3 (Bethesda). 2012 Dec 1;2(12):1541–54. doi: 10.1534/g3.112.004051 (PMC3516476; doi:10.1534/g3.112.004051)
Supplement: Supporting Information [file supp_2.12.1541_FigureS1.pdf]

A

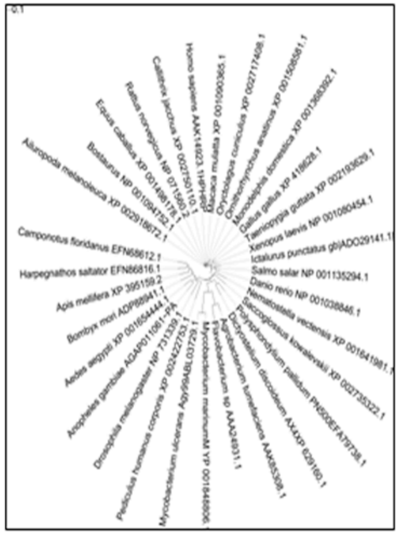

B

Bacteria strains	Location of *opd/opd*A gene	Geographical distribution	Phylogenetic Distribution (Class)	Accession Number of the sequence
Brevundimonas diminuta strain MG	Plasmid pCMS1	USA (Texas)	α-proteobacteria	HQ839670.1
Flavobacterium(Sphingomonas fuliginis) sp. ATCC 27551	Plasmid pPDL2	Philippines (IRRI)	α-proteobacteria	AJ421424.1
Flavobacterium balustinum	Plasmid pBC9	India (Anantapur)	Flavobacteria	AJ426431.1
Sphingomonas sp. JK1	Unknown	India (Mumbai)	α-proteobacteria	EU709764.1
Agrobacterium tumefaciens	Chromosomal	Australia	α-proteobacteria	AY043245.2

Figure S1 Geographical distribution of *opd* genes
