# Supplementary figures and images for: Multiple Mechanisms Contribute to Lateral Transfer of an Organophosphate Degradation (opd) Island in Sphingobium fuliginis ATCC 27551
Source: G3 (Bethesda). 2012 Dec 1;2(12):1541–54. doi: 10.1534/g3.112.004051 (PMC3516476; doi:10.1534/g3.112.004051)

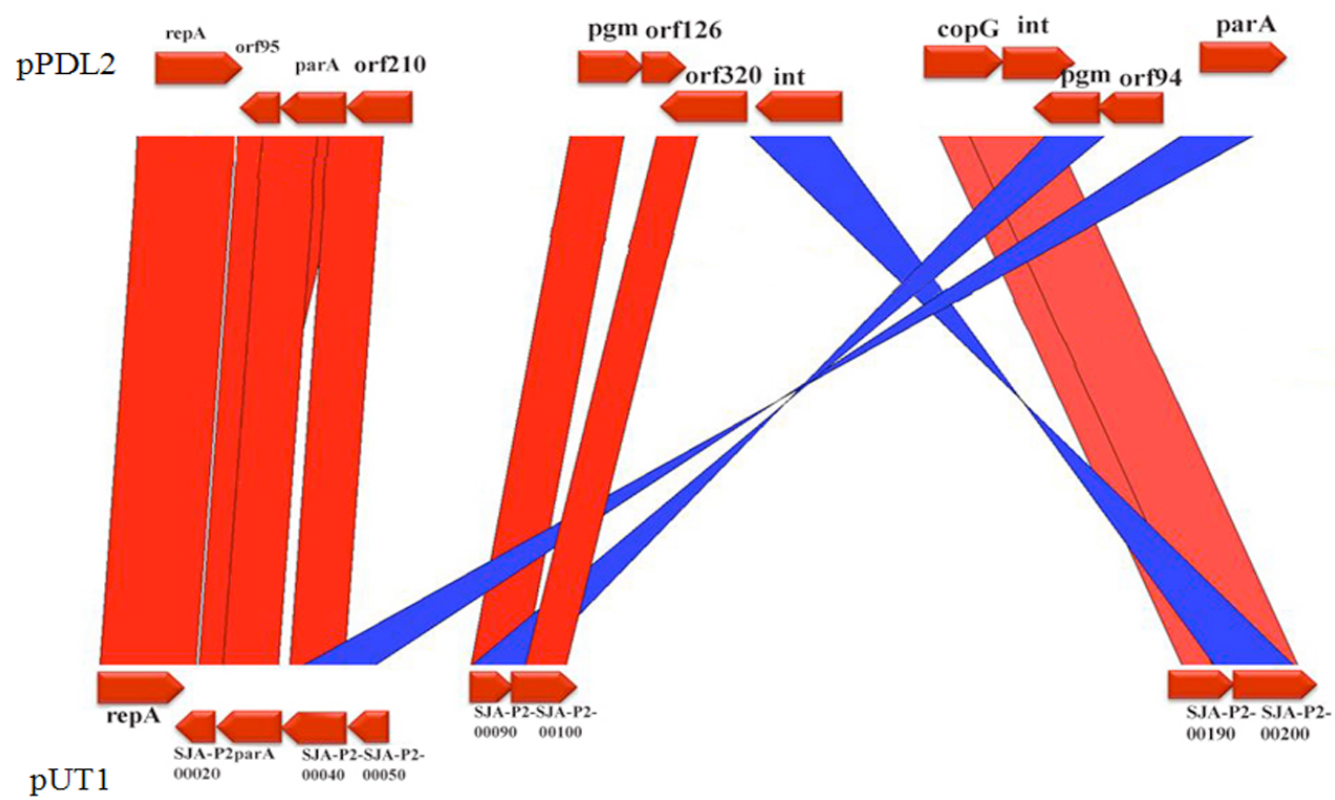

**Figure S2** Genetic map of *oriV* and *int* regions

Supplement: Supporting Information [file supp_2.12.1541_FigureS2.pdf]
