# Supplementary material for: Multiple Mechanisms Contribute to Lateral Transfer of an Organophosphate Degradation (opd) Island in Sphingobium fuliginis ATCC 27551
Source: G3 (Bethesda). 2012 Dec 1;2(12):1541–54. doi: 10.1534/g3.112.004051 (PMC3516476; doi:10.1534/g3.112.004051)
Supplement: Supporting Information [file supp_2.12.1541_TableS1.pdf]

Table S1 List of primers used in this study

Name of the primers	Sequences	Description
M13F	TGTAAAACGACGGCCAGT(F*)	Universal sequencing primers
M13R	GATAACAATTTCACACAGGA(R*)	
DSF0001	CGGAGCGGTGGCCGAGTGGTCGAAGGCGCTCGCCTGGAAAGTGAGTATACGTCAAAAGCG(F*)	Overlapping primers used to synthesize seryl *tRNA* gene with *att*B at 3' end
DSF0002	ATGGCGGAGCGGGAGGGATTCGAACCCTCGATACGCTTTTGACGTATACTCACTTTCCAG(R*)	
DSF0003	GAGAATTCCGGAGCGGTGGCCGAGTGGT(F*)	Primers used to amplify *att*B sequence
DSF0004	GAGAATTCATGGCGGAGCGGGAGGGATT(R*)	
DSF0005	TCTTGAGATATCACTGATAGATACAAGAGC(F*)	Primers used to amplify *bla*, *oriR101* and *repA101*^{ts} sequences from pKD46
DSF0006	CAATAACCCGGGTAAATGCTTCAATA(R*)	
DSF0007	CGCGAGCATCAGGCTTGG(F*)	Primers used for *att*PBL amplification from cointegrate
M13R	GATAACAATTTCACACAGGA(R*)	
DSF0008	GGGCATGCGGTCGTCATCCTTGTGCAT(R*)	Primers used for *att*BPR amplification from cointegrate

M13F	TGTAAAACGACGGCCAGT(F*)	
DSF0009<br>DSF0010	GCTGAGGTGGATCCATGAATCATGCAAC(F*)<br>AGATAGGTCTCGAGTGCTAGGCAGGCGCAGCG(R*)	Primers used to amplify *repB*
DSF0011<br>DSF0012	GGCTTGCTGCAGGCGTGAGCACACCTA(F*)<br>GGCTACTGCAGGCGTATACAGCTATAC(R*)	Primers used to amplify *oriV* and *repA* of pPDL2
DSF0013<br>DSF0014	CCGGACCATATGACATGGCCGCTGCC(F*)<br>GCGGGAAGCTTCAGTTCAGGTGGCG(R*)	Primers used to amplify *ligA* and *ligB*
DSF0015<br>DSF0016	CTGGCAGGAGGCGCAACTCA(F*)<br>CAAGGATGCTGTCTTTGACAACAGATG(R*)	Primers used to amplify *sac*B
DSF0017<br>DSF0018	CTGACAATCGAGGAGCACTACAC(F*)<br>CTTAGTTGCAGAAATAGGCGACCTT(R*)	Primers used for amplification of right and left ends of predicted Tn3 transposon

* F= Forward primer/ R= Reverse Primer
